# EEG dynamical features during variable-intensity cycling exercise in Parkinson’s disease

**DOI:** 10.3389/fnhum.2025.1571106

**Published:** 2025-04-28

**Authors:** Zahra Alizadeh, Emad Arasteh, Maryam S. Mirian, Matthew A. Sacheli, Danielle Murray, Silke Appel-Cresswell, Martin J. McKeown

**Affiliations:** ^1^Department of Electrical and Computer Engineering, University of Tehran, Tehran, Iran; ^2^Pacific Parkinson’s Research Centre, Djavad Mowafaghian Centre for Brain Health, University of British Columbia, Vancouver, BC, Canada; ^3^Division of Neurology, Faculty of Medicine (Neurology), University of British Columbia, Vancouver, BC, Canada

**Keywords:** EEG, pedaling, Parkinson’s, Catch-22 features, MCCA

## Abstract

**Background:**

Exercise is increasingly recognized as a beneficial intervention for Parkinson’s disease (PD), yet the optimal type and intensity of exercise remain unclear. This study investigated the relationship between exercise intensity and neural responses in PD patients, using electroencephalography (EEG) to explore potential neural markers that could be ultimately used to guide exercise intensity.

**Method:**

EEG data were collected from 14 PD patients (5 females) and 8 healthy controls (HC) performing stationary pedaling exercises at 60 RPM with resistance adjusted to target heart rates of 30, 40, 50, 60, and 70% of maximum heart rate. Subjects pedaled for 3 min at each intensity level in a counterbalanced order. Canonical Time-series Characteristics (Catch-22) features and Multi-set Canonical Correlation Analysis (MCCA) were utilized to identify common profiles of EEG features at increasing exercise intensity across subjects.

**Results:**

We identified a statistically significant MCCA component demonstrating a monotonic relationship with pedaling intensity. We have discovered nine features which show significant trends across intensity (*p*-value<0.01), and the dominant feature in this component was Periodicity Wang (*p*-value<0.0001), related to the autocorrelation of the EEG. Analysis revealed a consistent trend across features: six features increased with intensity, indicating heightened rhythmic engagement and sustained neural activation, while three features decreased, suggesting reduced variability and enhanced predictability in neural responses. Notably, PD patients exhibited more rigid, consistent response patterns compared to healthy controls (HC), who showed greater flexibility and variability in their neural adaptation across intensities.

**Conclusion:**

This study highlights the feasibility of using EEG-derived features to track exercise intensity in PD patients, identifying specific neural markers correlating with varying intensity levels. PD subjects demonstrate less inter-subject variability in motor responses to increasing intensity. Our results suggest that EEG biomarkers can be used to assess differing brain involvement with the same exercise of increasing intensity, potentially useful for guiding targeted therapeutic strategies and maximizing the neurological benefits of exercise in PD.

## 1 Introduction

PD is a neurodegenerative disease associated with the accumulation of alpha-synuclein and loss of dopamine-producing neurons, particularly in the substantia nigra (Calabresi et al., 2023). Symptoms of this dopaminergic neuron loss include bradykinesia, muscle rigidity, and tremors, in addition to a host of non-motor symptoms (Licen et al., 2022). Current treatments such as medication and deep brain stimulation (DBS) can alleviate its symptoms but do not fundamentally alter the course of the disease and vary in effectiveness depending on the individual (Ridgel et al., 2015). Consequently, it is crucial to explore new therapeutic approaches which can have rehabilitation potential without side effects.

Numerous studies have investigated the potential of exercise in improving symptoms of various neurological problems including neurodegenerative diseases such as Parkinson’s disease (Langeskov-Christensen et al., 2024), and Alzheimer’s (Valenzuela et al., 2020). Valenzuela et al. (2020) shed light on the advantageous role of regular physical exercise [Aerobic exercise (with an intensity of 50–75% of VO₂ max)] in mitigating Alzheimer’s disease hallmarks, hippocampal volume reduction, spatial memory decline, and learning impairments in Alzheimer’s disease. Exercise has also had a positive impact on balance in patients with stroke, PD, and multiple sclerosis [for a systematic review see: Salari et al. (2022)]. Exercise has also been beneficial in psychiatric diseases such as anxiety (Pontifex et al., 2021), and Obsessive Compulsive Disorder (OCD) (Abrantes et al., 2019) with moderate-intensity aerobic exercise. In a meta-analysis (Ensari et al., 2015), showed that acute exercise sessions provide immediate relief from state anxiety, and Pontifex et al. (2021) demonstrated its ability to alleviate cognitive impairments associated with anxiety.

Cycling appears to be a particularly beneficial exercise for individuals with PD. Alberts et al. (2011) noted improvements in a person living with Parkinson’s disease (PwP) within two days after a tandem cycling expedition. Follow-up studies (Ridgel et al., 2009; Ridgel et al., 2012; Ridgel et al., 2015) revealed significant benefits, including a 38% reduction in tremors and a 28% improvement in bradykinesia after eight weeks of forced tandem cycling. Dynamic high-cadence cycling also showed motor improvements, while other research (Snijders et al., 2012; Licen et al., 2022) highlighted the persistence of cycling ability in more advanced disease, even in those with severe freezing of gait. The repetitive nature of cycling poses a lower physical challenge and may help stabilize abnormal neural oscillations, making it a promising rehabilitative option for PD patients (Licen et al., 2022). In addition, Recent evidence suggests that integrating virtual reality and exergaming into rehabilitation programs may further enhance motor and cognitive outcomes in PD. A systematic review by (Marotta et al., 2023) found that exergaming-based interventions, particularly those incorporating virtual reality, improve balance, executive function, and motor performance, making them a promising adjunct to traditional exercise therapies.

Despite the well-documented beneficial effects of exercise on brain function, there are still significant gaps in understanding how specific factors such as the duration, type, and intensity of exercise influence these outcomes. Most studies have focused on comparing pre- and post-exercise brain states (Moraes et al., 2007; Gutmann et al., 2015). However, monitoring EEG *during* exercise could provide valuable insights into how these variables interact (Enders et al., 2016; Bailey et al., 2008), potentially enhancing our ability to optimize exercise-based therapeutic interventions. This study aims to address these gaps by exploring how dynamic brain activity during exercise—particularly through EEG—can provide deeper understanding of exercise-induced neurophysiological changes.

Recent research has focused on leveraging dynamic features from brain recordings like EEG to better understand neurodegenerative diseases (Cacciotti et al., 2024). EEG has emerged as a promising biomarker for tracking disease progression and assessing the neurophysiological effects of rehabilitation in PD. Studies have shown that alterations in EEG power bands features correlate with motor deficits, providing valuable insights into disease severity and treatment efficacy (Miladinović et al., 2021). These features offer critical insights into the temporal fluctuations of brain connectivity, which may assist in interpreting symptom progression, and mechanisms behind cognitive and motor impairments. Lubba et al. (2019) proposed 22 key temporal features, known as Canonical Time-series Characteristics (Catch-22), from an initial set of 4,791. These features effectively capture diverse aspects of temporal dynamics of various time series, including EEG, that capture extreme events, autocorrelation, symbolic patterns, and scaling behaviors, while reducing redundancy, making them broadly applicable across time-series analyses.

The main goal of this study is to explore how different pedaling intensities impact brain activity in individuals with Parkinson’s disease (PD). We will look at EEG data to track neural responses as exercise intensity changes. We believe that higher pedaling intensity will lead to clear changes in brain activity, which we can identify using Catch-22 features. In this study, we applied Catch-22 dynamic EEG features to assess the effects of pedaling intensity on brain activity in individuals with PD and healthy controls. Participants pedaled at five different intensity levels while their EEG data were collected. Using MCCA (Fu et al., 2019), we identified linear combinations of EEG channels that exhibited consistent trends across participants, highlighting key neural responses as pedaling intensity increased.

## 2 Methods

### 2.1 Experimental protocol

In this study, 14 PD and 8 healthy control (HC) subjects were recruited from the Movement Disorders Clinic at the Pacific Parkinson’s Research Centre, Vancouver, Canada. All subjects provided written, informed consent, and all research was reviewed and approved by the appropriate Ethics Boards. Disease severity was assessed using the Hoehn and Yahr scale (stages 1–3), and symptom severity was quantified using the Unified Parkinson’s Disease Rating Scale (UPDRS), with a mean score of 18.96 (SD = 10.43). Additionally, all PD participants were in the early stages of the disease and regularly engaged in aerobic exercise (defined as >100 min of moderate aerobic activity per week over the last three months). To optimize exercise tolerance, subjects took their usual anti-parkinsonian medication. Exclusion criteria included a history of stroke, severe cardiovascular disease, or any other significant neurological or psychiatric disorder that could affect motor or cognitive function. Individuals with severe musculoskeletal impairments preventing safe participation in cycling exercises were also excluded. More information about PD subjects is in Table 1 in the results section.

**Table 1:** Demographic information about the subjects included in this study.

Variables	Values
Age	60.66 ± 7.81
Male/Female	9/5
Hoehn-Yahr	1–3
UPDRS	18.96 ± 10.43
Regular exercise	>100 min per week over 3 months
Montreal cognitive assessment score	>24 (27.53 ± 1.92)
Beck’s depression score	<13 (2.27 ± 3.63)
Beck’s anxiety score	<9 (3.67 ± 3.2)
Fatigue severity scale (FSS)	20.21 ± 10.25
Positive and negative affect schedule (PANAS)	28.42 ± 5.74
Trial Making Test (TMT) A	30.57 ± 7.98
TMT B	63.81 ± 32.72
Medication	Beta-blocker “Off” Anti-Parkinson “On”
Handedness (Right/Left)	11/4

Participants underwent standardized cognitive and mood evaluations before the experimental session. Cognitive status was screened using the Montreal Cognitive Assessment (MoCA), with all participants scoring above 24 (mean = 27.53, SD = 1.92). Psychological well-being was evaluated using the Beck Depression Inventory (BDI) and the Beck Anxiety Inventory (BAI), with mean scores of 2.27 (SD = 3.63) and 3.67 (SD = 3.2), respectively. Fatigue was assessed using the Fatigue Severity Scale (FSS) (mean = 20.21, SD = 10.25), and emotional affect was measured using the Positive and Negative Affect Schedule (PANAS) (mean = 28.42, SD = 5.74). Additionally, cognitive processing speed and executive function were evaluated using the Trail Making Test (TMT), with a mean completion time of 30.57 s (SD = 7.98) for TMT A, assessing visual attention and processing speed, and 63.81 s (SD = 32.72) for TMT B, which measures cognitive flexibility and executive function.

Participants pedaled on a stationary bicycle, with the seat height individually adjusted for comfort. Each session began with a 5-min warm-up to ensure safety. Subjects pedaled at intensities of 30, 40, 50, 60, and 70% of their maximum heart rate [calculated as 0.85 * (220 – age)] while maintaining a steady pace of 60 RPM. Resistance was adjusted to vary intensity, with 3-min intervals followed by 3-min recovery periods. Intensity intervals were pseudo-randomly assigned, except for 70%, which never came first. The 30-min intervention concluded with a 5-min cool-down.

EEG was recorded using nine electrodes at standard locations F3, Fz, F4, C3, Cz, C4, P3, Pz, and P4, targeting frontal, central, and parietal regions critical for monitoring brain activity during exercise. A stretchable head cap with Ag-AgCl electrodes (ANT Wave Guard cap, Advanced Neuro Technology B.V.) was used, with the reference electrode placed near the vertex between Cz and CPz. The ground electrode was positioned between Fz and FPz on the frontal scalp, and electrode impedances were maintained below 10 kΩ to ensure signal quality. The EEG recording was performed continuously, beginning with the warm-up phase (first three minutes) and concluding after the cool-down phase (final three minutes). Importantly, the recording remained uninterrupted during rest stages between pedaling sessions, ensuring the continuous capture of neural activity throughout the experiment.

### 2.2 Preprocessing

We performed a number of careful steps to remove the artifact from the EEG. First, the common average reference (CAR) technique was applied, followed by high pass FIR filtering, low pass FIR filtering, and notch filtering to remove frequencies below 0.1, above 100, and 60 Hz line noise. A recursive least squares (RLS) algorithm was applied to remove eye-related (ocular) artifacts, and an adaptive filter was used in this method to remove eye blinks from the EEG signal by adjusting its coefficients in each iteration according to the difference between the EEG signal and the EOG signal. The automatic artifact rejection (AAR) plugin was used in EEGLAB (MATLAB 2018b) to reject this artifact. Then, the EMG artifact was removed with principal component analysis (PCA). In the last step, another remaining artifact source was removed with the help of the IClabel plugin of EEGLAB, with two of the nine components removed.

### 2.3 Feature extraction using Catch-22

We utilized the Catch-22 package, which allows for the extraction of 22 distinct features from each EEG channel, as listed in Table S1. The duration for feature extraction was specifically set to one second, corresponding to one complete cycle of pedaling (60 RPM). By synchronizing the feature extraction window with the pedaling cycle, we effectively isolated and minimized the impact of pedaling frequency on the EEG signals, ensuring that the extracted features more accurately reflected the underlying brain activity rather than the mechanical motion.

### 2.4 MCCA-based extraction of intensity-linked neural signatures

**Figure 1:**
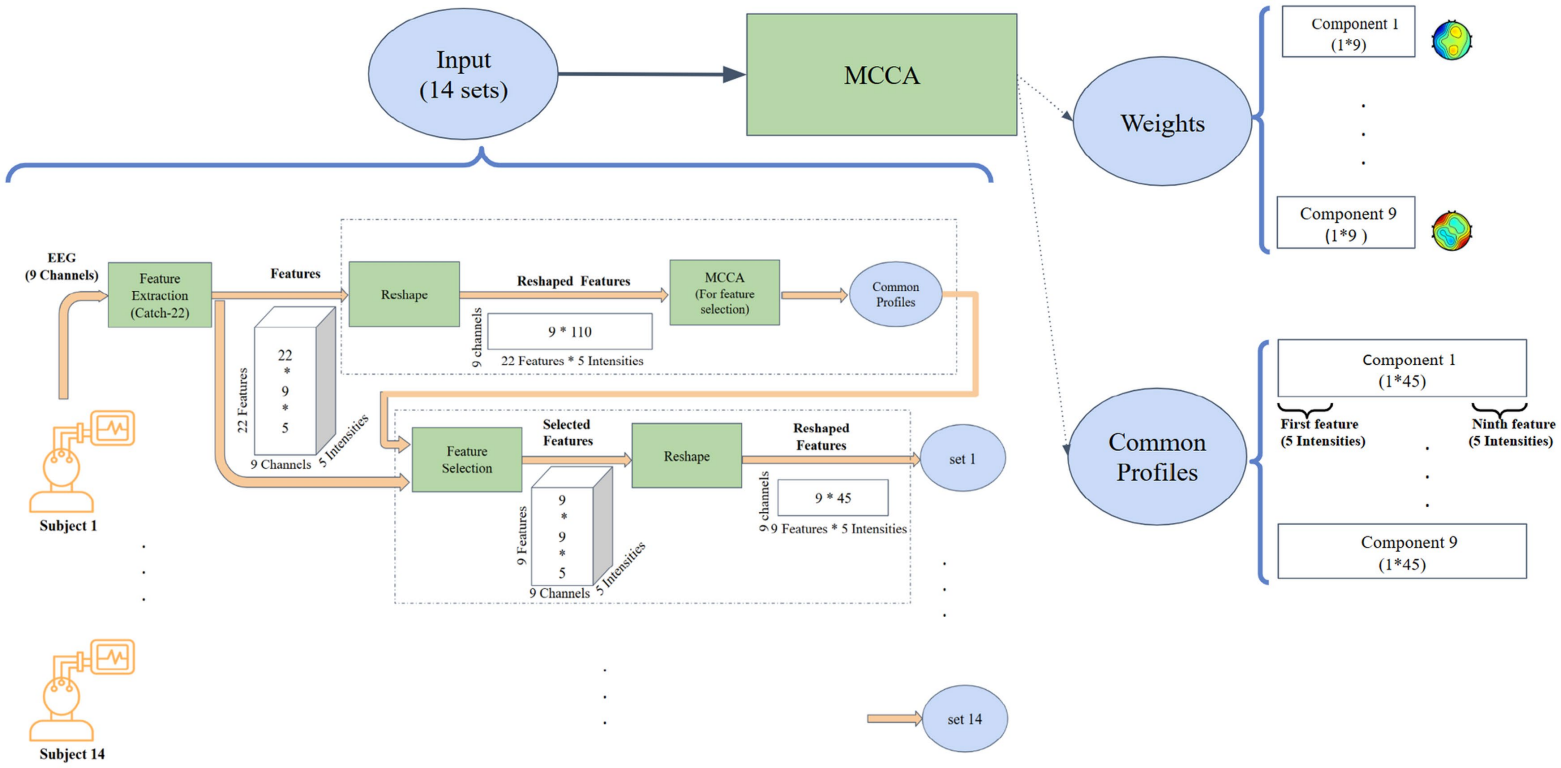
The pipeline displays the identification process of the most consistent features across EEG channels, highlighting those with the strongest relationship to exercise intensity.

MCCA extends traditional CCA by analyzing relationships across multiple datasets. It identifies optimal linear combinations of variables (canonical variates) that maximize correlations between datasets. In this study, MCCA was applied to EEG data from 14 participants in a two-step process (Figure 1).

#### 2.4.1 First round: feature selection

In the first round (upper dashed rectangle in Figure 1), the data matrix for each subject was structured as 9 EEG channels by 110 observations (22 features across five intensity levels). This round served as a feature selection step, aiming to identify features that consistently represented shared neural dynamics across EEG channels of all subjects. Nine canonical components were extracted (corresponding to the number of EEG channels), and features with the highest consistency across subjects were identified for further analysis.

#### 2.4.2 Second round: detecting neural signatures

Building on the results above, the second round of MCCA focused on detecting the neural signatures of exercise intensity. Informative features selected from the first round, evaluated for their consistency across subjects within components 2–4 (as detailed in the results section), were used to refine the data matrices. Feature selection was performed using Repeated-Measures ANOVA (RM-ANOVA), applied to components 2, 3, and 4 to assess feature consistency across subjects. Features that exhibited *p*-values < 0.001 in 2–4 components were considered stable and selected for further analysis. These matrices, structured as 9 channels by 45 observations (9 features across 5 intensities), enabled more targeted analysis. This two-step approach ensured improved interpretability and computational efficiency by concentrating on features most relevant to exercise-induced neural dynamics.

### 2.5 Weighted signal analysis and wavelet transformation

To integrate the multichannel EEG data, a weighted aggregate signal was computed using channel weights derived from the MCCA analysis. To capture critical events in both the time and frequency domains, the Wavelet Transform was employed. Before transformation, EEG signals were averaged over one-second windows (corresponding to a single pedaling cycle at 60 RPM) to minimize noise and artifacts. This averaging step ensured that the signals reflected underlying neural activity rather than transient noise or mechanical motion.

## 3 Results

Table 1 summarizes the demographic and baseline clinical characteristics of the study participants. The mean age was 60.66 years (SD = 7.81), with a gender distribution of 9 males and 5 females. Participants demonstrated mild to moderate symptom severity based on Hoehn-Yahr staging (1–3) and a mean UPDRS score of 18.96 (SD = 10.43). Cognitive and psychological assessments indicated overall preserved cognitive function and low levels of depression and anxiety. Participants also reported regular engagement in aerobic exercise and demonstrated relatively good functional and emotional status, as reflected in the FSS, PANAS, and TMT scores.

### 3.1 Artifact identification via principal component analysis

**Figure 2:**
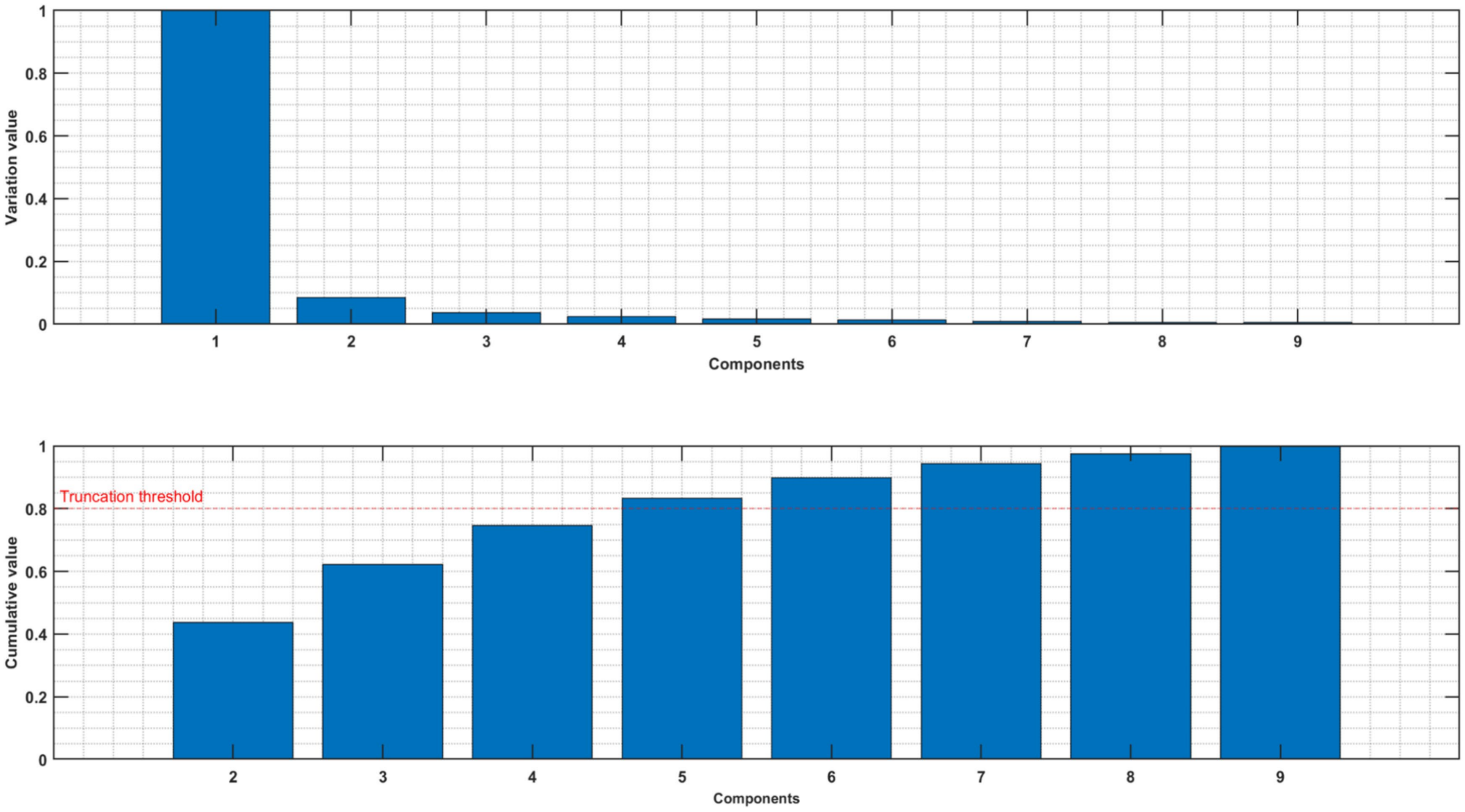
Upper panel: normalized variance of each component of MCCA. Lower panel: normalized cumulative variance of components 2–9.

Consistent with prior studies (De Cheveigné et al., 2019) the first component of MCCA may still relate to residual artifacts (Jiang et al., 2019) as illustrated in Figure 2 for one subject. We therefore excluded the first component and concentrated our further investigations on components 2–4.

### 3.2 MCCA-based extraction of intensity-linked neural signatures

**Figure 3:**
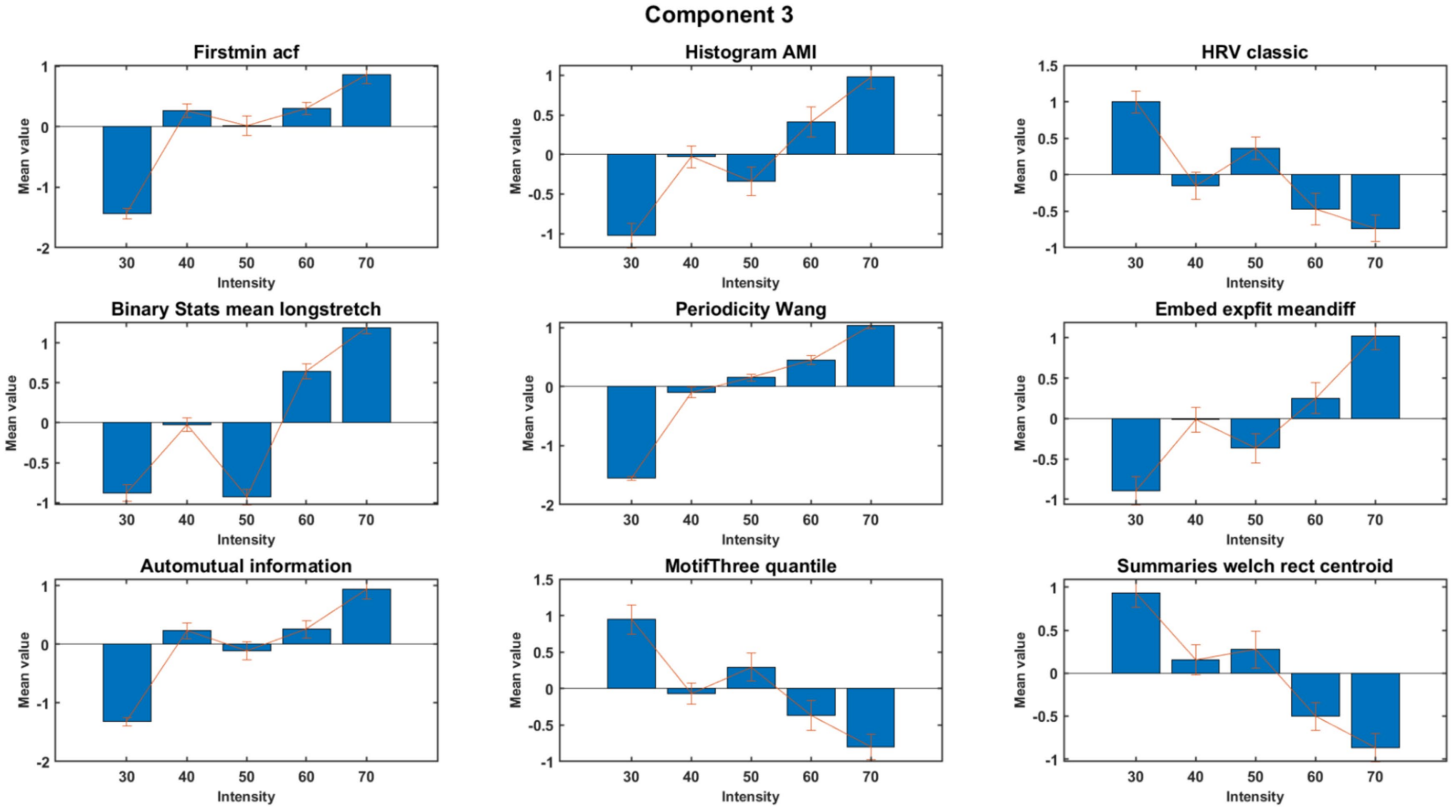
Trend across intensity for features of component 3 of PD subjects. The figure shows the aggregated (normalized) mean and standard deviation across subjects.

**Figure 4:**
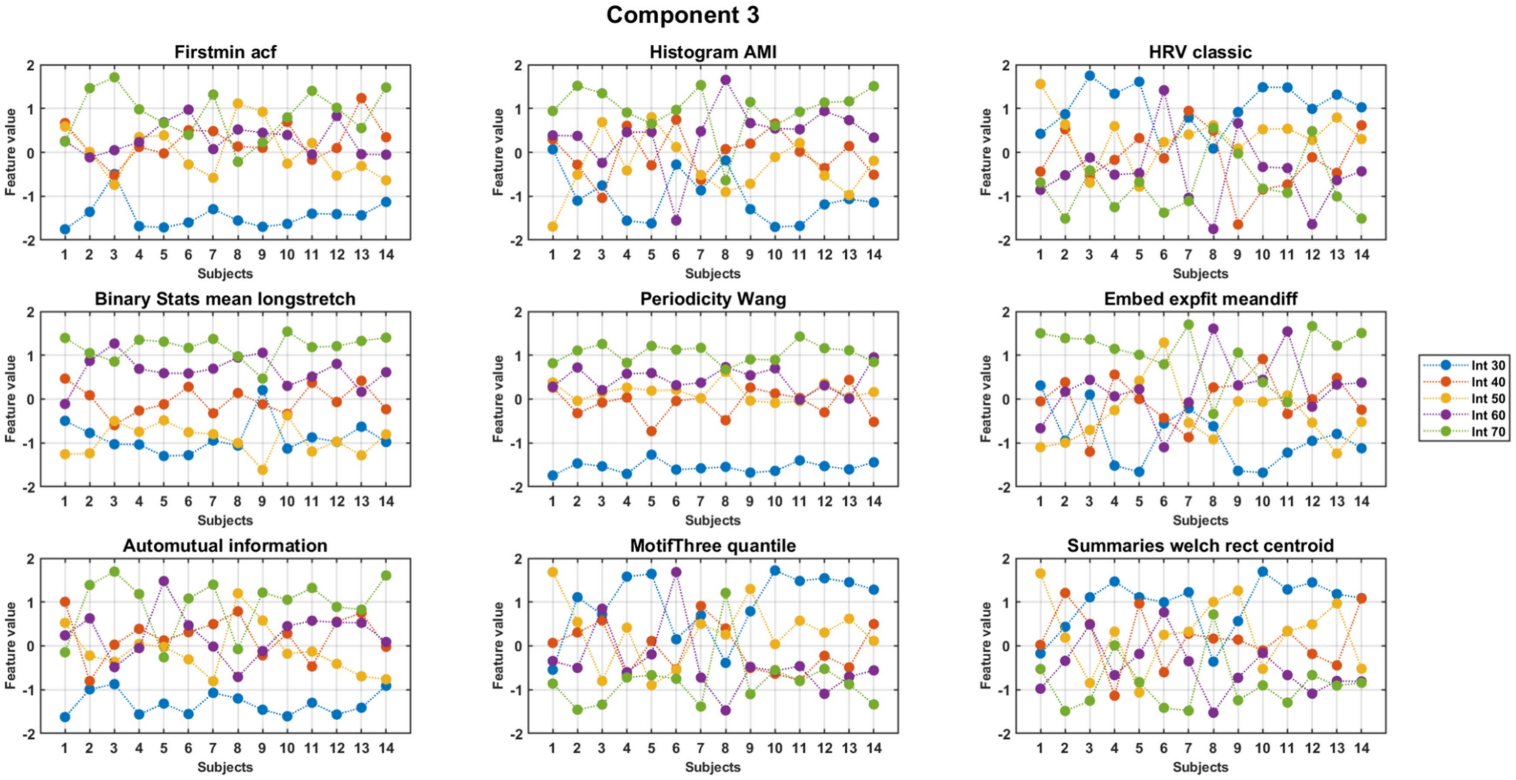
Trends across intensity for features of component 3 of PD subjects. The figure displays (normalized) values of features for individual subjects (*x*-axis) for each feature.

MCCA analysis at the first round demonstrated nine features that exhibited high consistency across subjects within the meaningful components 2, 3, and 4. The feature values were normalized separately to obtain a better visual representation. Nine out of the 22 features exhibited consistent behavior across subjects. A detailed description of these nine consistent features is presented in Table 2. The results for component 3 are shown in Figures 3, 4, with Supplementary Figure S1 showing the control group for comparison. Six features, as mentioned in the second column of Table 2, showed an increasing trend with rising exercise intensity, while three features showed a decreasing trend.

**Table 2:** Most consistent features of the Catch-22 package.

Feature no.	Feature name	Category	Direction	Description	Justification for relevance to pedaling effects
4	CO_FirstMin_ac	Linear autocorrelation	Increasing	Lag of the first minimum of the autocorrelation function	The first moment of decreasing signals self similarity, detecting changes in brain activity due to rhythmic pedaling
5	CO_HistogramAMI_even2_5	Nonlinear autocorrelation	Increasing	Dependency between successive points in the time series	The extent of information sharing over intervals, useful for analyzing regular, cyclical motion impacts
7	MD_hrv_classic_pnn40	Successive difference	Decreasing	The proportion of pairs of successive differing more than 4% of standard deviation (a classic measure in heart rate variability studies)	Changes induced by physical exertion from pedaling
8	SB_BinaryStats_mean_longstretch1	Simple temporal statistics	Increasing	Length of the longest sequence above the mean, indicative of sustained higher activity levels	Prolonged periods of high brain activity, potentially induced by consistent pedaling
10	PD_PeriodicityWang_th0_01	Others	Increasing	The regularity and periodicity in data	Directly relevant to detecting rhythmic patterns in EEG signals correlated with pedaling cadence
11	CO_Embed2_Dist_tau_d_expfit_meandiff	Successive difference	Increasing	Goodness of exponential fit to the time series, capturing the average change over 2D embedded dimensions	Useful for understanding the adaption of brain dynamics over time during continuous pedaling
12	IN_AutoMutualInfoStatS_40_gaussian_fmmi	Nonlinear autocorrelation	Increasing	The first minimum of auto mutual information, reflecting the least predictable Point in time	Moment of maximum change in brain state, triggered by variations in pedaling speed or intensity
18	SB_MotifThree_quantile_hh	Successive difference	Decreasing	Shannon entropy of three-symbol motifs, assessing the predictability and complexity of patterns	Effective in capturing the complexity of brain signal responses to repetitive physical activities like pedaling
22	FC_LocalSimple_mean3_stderr	Linear autocorrelation	Decreasing	Standard error of a local simple mean with a window of three, assessing prediction error in the short-term	predictability of brain activity during physical exercise

Feature numbers and names are mentioned based on numbers listed in Lubba et al. (2019).

Moreover, by focusing on the weighted vector of EEG data computing by multiplying EEG by the weights of MCCA, we observed a distinct cluster of harmonics that was concentrated around the alpha band, as depicted in the supplementary section in Supplementary Figure S4.

### 3.3 Post-hoc analysis: investigation of the difference between PD and HC

To evaluate differences in pedaling intensity and compare individuals with PD to healthy controls (HC), we conducted a repeated measures Analysis of Variance (ANOVA). We examined both the within-subject factor of pedaling intensity and the between-subject factor of groups (PD vs. HC) to understand how features vary under different conditions. Results showed significant intensity interactions within the PD group (10.25 < *F* < 514.5, 0 < *p* < 0.004) and significant differences between groups (8.79 < *F* < 533.77, 0 < *p* < 0.007). HC subjects exhibited greater variability across intensities, while PD participants showed more consistent, predictable feature changes. Details are provided in Supplementary Table S2.

## 4 Discussion

This study offers new insights into the link between exercise intensity and brain dynamics in PD, highlighting distinct neural activity patterns during pedaling. The key finding is the identification of consistent EEG features, derived through MCCA, that reliably track changes in pedaling intensity. These measurable and robust neural responses across subjects present promising potential biomarkers for tailoring and optimizing exercise-based interventions for PD management. Recent research has also highlighted the potential of EEG-based biomarkers in PD, demonstrating their role in tracking disease progression and evaluating treatment efficacy (De Laat et al., 2024).

By applying the MCCA to identify consistent Catch-22 EEG features across subjects, we allowed individual channel combinations to vary from subject-to-subject. Six features in Component 3 increased with intensity (FirstMin_acf, HistogramAMI_even_25, BinaryStats_meanlongstretch, PeriodicityWang, Embed2_Dist_expfit_meandiffand and AutoMutualInfoStats), reflecting enhanced rhythmic coordination, prolonged engagement, and adaptive responses. Conversely, three features (Hrv_classic, MotifThree_quantile, and LocalSimple_mean3stderr) decreased, indicating reduced variability and increased neural efficiency, aligning with the demands of higher-intensity exercise. These results align with prior work indicating that increased exercise intensity influences neural oscillations, in the alpha frequency range (Ciria et al., 2018). Prior work has shown that cycling has been shown to both suppress beta-band local field potentials from Deep Brain Stimulation electrodes but also enhance narrowband power increases around 18 Hz (Storzer et al., 2017). Additionally, recent findings suggest that different intensities of aerobic exercise can modulate key neurotransmitter levels in PD, such as dopamine, norepinephrine, and serotonin, which could contribute to enhanced executive function and motor control (Tsai et al., 2024).

We observed reduced variability and flexibility in neural responses to increasing exercise intensity in individuals with PD. In PD, beta-band oscillations are excessively synchronized, with more consistent waveform features, such as sharper peaks and greater steepness asymmetry, compared to healthy controls (Jackson et al., 2019). In PD patients with Freezing of Gait, higher EEG amplitude synchronization across frequency bands (theta, alpha, beta, and gamma) has been observed between different brain regions, regardless of the motor task (Asher et al., 2021). These observations suggest that subjects with PD have impaired metastability, a critical property of the brain’s dynamics. Metastability reflects the brain’s ability to balance stability and flexibility, supporting efficient information processing, cognitive function, and adaptability. In the context of exercise, it enables the brain to transition between varying intensity levels, expanding its dynamic repertoire (Bapat et al., 2024) and supporting the functional states needed for diverse motor and cognitive activities. Growing evidence suggests that exercise-induced neuroplasticity plays a key role in these adaptations, as sustained physical activity has been shown to promote structural and functional brain changes that enhance both motor and cognitive performance in PD (Langeskov-Christensen et al., 2024).

In this research, novel use of EEG biomarkers have been employed to quantify the real-time impact of exercise intensity on brain activity in PD. This method, unlike conventional measures, provides an objective and dynamic quantification of neural responses, which could be valuable for disease monitoring and optimizing rehabilitation. Additionally, this research utilizes a data-driven approach with MCCA, allowing for the extraction of reliable EEG features that signal physiological responses to exercise. This method holds promise for clinical use in the future, whereby personalized exercise prescriptions could be optimized based on neural responses, ultimately maximizing the outcome for motor function and quality of life for individuals with PD.

Future research should explore whether these acute neural changes are reflected in long-term functional benefits and whether specific exercise intensities can be optimized to induce neural plasticity in PD. Additionally, the incorporation of EEG-based monitoring in the clinical environment could optimize rehabilitation protocols and provide more personalized approaches to exercise therapy for PD patients.

This study only evaluates the acute effects of exercise on brain activity rather than its long-term benefits. While our findings suggest that exercise intensity influences neural dynamics in PD, future research is needed to determine whether these neural adaptations persist over time and contribute to sustained motor and cognitive improvements. Additionally, the relatively small sample size limits the generalizability of our findings, highlighting the need for more extensive studies to validate these EEG-based biomarkers further and their potential role in guiding individualized exercise interventions for PD.

Nevertheless, this study has certain limitations that should be considered. Our analysis primarily relied on linear methods, such as MCCA, which may only partially capture the complexity of neural responses during exercise. Future studies should explore nonlinear approaches to better detect exercise-induced neural changes and uncover subtler patterns of brain activity. It is worth noting, however, that the Catch-22 package already includes several nonlinear measures, which offer a valuable starting point for these investigations. Also, the sample size of the study is relatively small, which may limit the generalizability of the findings and the strength of the conclusions drawn. Additionally, the use of only nine EEG electrodes limited the spatial resolution of the recorded brain activity, suggesting that future research could benefit from higher-density EEG arrays or multimodal neuroimaging techniques, such as combining EEG with fMRI. Further research could also assess the consistency of the identified features across varying pedaling intensities in a larger cohort, ensuring their robustness and relevance to motor performance and clinical outcomes. The potential of the Periodicity Wang feature as a biomarker could be explored using other modalities, such as fMRI and ECG, to determine its broader applicability across neural and physiological contexts.

## 5 Conclusion

This study highlights the potential of EEG-derived features to monitor and assess exercise intensity in PD patients. Among these, the Periodicity Wang (PW) feature, which measures the autocorrelation of neural dynamics, showed a strong association with changes in pedaling intensity. By applying Catch-22 features and MCCA, we observed consistent neural responses across subjects at different intensity levels. Some features exhibited increasing trends, indicating enhanced rhythmic coordination and sustained neural activation, while others displayed decreasing trends, reflecting reduced variability and a transition to more stable, predictable neural patterns. These findings offer valuable insights for optimizing exercise-based interventions in PD and highlight the potential for identifying neural biomarkers to guide future therapeutic strategies.

## Data Availability

The GitHub repository, including the codes of this paper, can be accessed here: https://github.com/zahraalz3523/EEG-dynamical-features-. All data produced in the present study are available upon request to the authors.
