# Supplementary material for: EEG dynamical features during variable-intensity cycling exercise in Parkinson’s disease
Source: Front Hum Neurosci. 2025 Apr 28;19:1571106. doi: 10.3389/fnhum.2025.1571106 (PMC12066486; doi:10.3389/fnhum.2025.1571106)
Supplement: Supplementary file 1 [file Data_Sheet_1.docx]

# Supplementary

Supplementary 1: A description of all Catch-22 features is brought in Table S1.

Table S1: Features and interpretation of Catch-22 features [(Lubba et al., 2019)](https://www.zotero.org/google-docs/?tVeFNj)

| **Feature No.** | **Feature name** | **Category** | **Description** | **Justification for Relevance to Pedaling Effects** |
| --- | --- | --- | --- | --- |
| 1 | DN_HistogramMode_5 | Distribution shape | mode of 5-bin histogram distribution | The most z-scored distributed value |
| 2 | DN_HistogramMode_10 | Distribution shape | mode of 10-bin histogram distribution | The most z-scored distributed value |
| 3 | CO_f1ecac | Linear  autocorrelation | Lag of the first 1/e crossing in the autocorrelation  function | The time constant of decay in self-similarity |
| 4 | CO_FirstMin_ac | Linear  autocorrelation | Lag time to the first minimum of the autocorrelation function | The first moment of decreasing signals self similarity, detecting changes in brain activity due to rhythmic pedaling |
| 5 | CO_HistogramAMI_even2_5 | Nonlinear  autocorrelation | Dependency between successive points in the time series | The extent of information sharing over intervals, useful for analyzing regular, cyclical motion impacts |
| 6 | CO_trev_1_num | Nonlinear  autocorrelation | The average across the cube of time series  successive difference | Time reversibility |
| 7 | MD_hrv_classic_pnn40 | Successive  difference | The proportion of pairs of successive differing more than 4% of standard deviation (a classic measure in heart rate variability studies) | Changes induced by physical exertion from pedaling |
| 8 | SB_BinaryStats_mean_longstretch1 | Simple temporal statistics | Length of the longest sequence above the mean, indicative of sustained higher activity levels | Prolonged periods of high brain activity, potentially induced by consistent pedaling |
| 9 | SB_TransitionMatrix_3ac_sumdiagcov | Others | Trace of covariance of transition matrix between 3 symbols | Temporal evolution of quantized neural activity |
| 10 | PD_PeriodicityWang_th0_01 | Others | The regularity and periodicity in data | Directly relevant to detecting rhythmic patterns in EEG signals correlated with pedaling cadence |
| 11 | CO_Embed2_Dist_tau_d_expfit_meandiff | Successive difference | Goodness of exponential fit to the time series, capturing the average change over 2D embedded dimensions | Useful for understanding the adaption of brain dynamics over time during continuous pedaling |
| 12 | IN_AutoMutualInfoStatS_40_gaussian_fmmi | Nonlinear  autocorrelation | The first minimum of auto mutual information, reflecting the least predictable Point in time | Moment of maximum change in brain state, triggered by variations in pedaling speed or intensity |
| 13 | FC_LocalSimple_mean1_tauresrat | Successive difference | Change in autocorrelation time scale  after iterative differencing | The ratio of stationarity in the signal compared to its first-order moment within the brain dynamics varies with intensity. |
| 14 | DN_OutlierInclude_p_001_mdrmd | Simple  temporal  statistics | Positive outlier timing | Distribution of over-threshold values in time |
| 15 | DN_OutlierInclude_n_001_mdrmd | Simple  temporal  statistics | Negative outlier timing | Distribution of under-threshold values in time |
| 16 | SP_Summaries_welch_rect_area_5_1 | Linear autocorrelation | Relative Power of lowest  20% frequencies | Distribution of low frequency power in EEG |
| 17 | SB_BinaryStats_diff_longstretch0 | Successive difference | Longest stretch of decreasing values | The longest time of nonstop decreasing in signal  values |
| 18 | SB_MotifThree_quantile_hh | Successive difference | Shannon entropy of three-symbol motifs, assessing the predictability and complexity of patterns | Effective in capturing the complexity of brain signal responses to repetitive physical activities like pedaling |
| 19 | SC_FluctAnal_2_rsrangefit_50_1_logi_prop__r1 | Fluctuation analysis | Rescaled range fluctuation analysis  (low-scale scaling) | The relative time of change in regime in the  multifractal signals |
| 20 | SC_FluctAnal_2_dfa_50_1_2_logi_prop_r1 | Fluctuation analysis | Detrended fluctuation analysis  (low-scale scaling) | The relative time of change in regime in the  multifractal signals |
| 21 | SP_Summaries_welch_rect_centroid | Linear autocorrelation | Centroid frequency | Distribution of power of frequencies |
| 22 | FC_LocalSimple_mean3_stderr | Linear autocorrelation | Standard error of a local simple mean with a window of three, assessing prediction error in the short term | predictability of brain activity during physical exercise |

Supplementary 2: We also applied MCCA to the control group to establish a baseline representation of the common patterns within a healthy population. This analysis enables a direct comparison with the PD group, allowing us to identify distinct differences and shared features between the two groups. By doing so, we can better understand the variations introduced by pathological conditions and ensure that our findings are robust and grounded in meaningful contrasts. The results of this analysis for the control group are brought in Fig. S1.


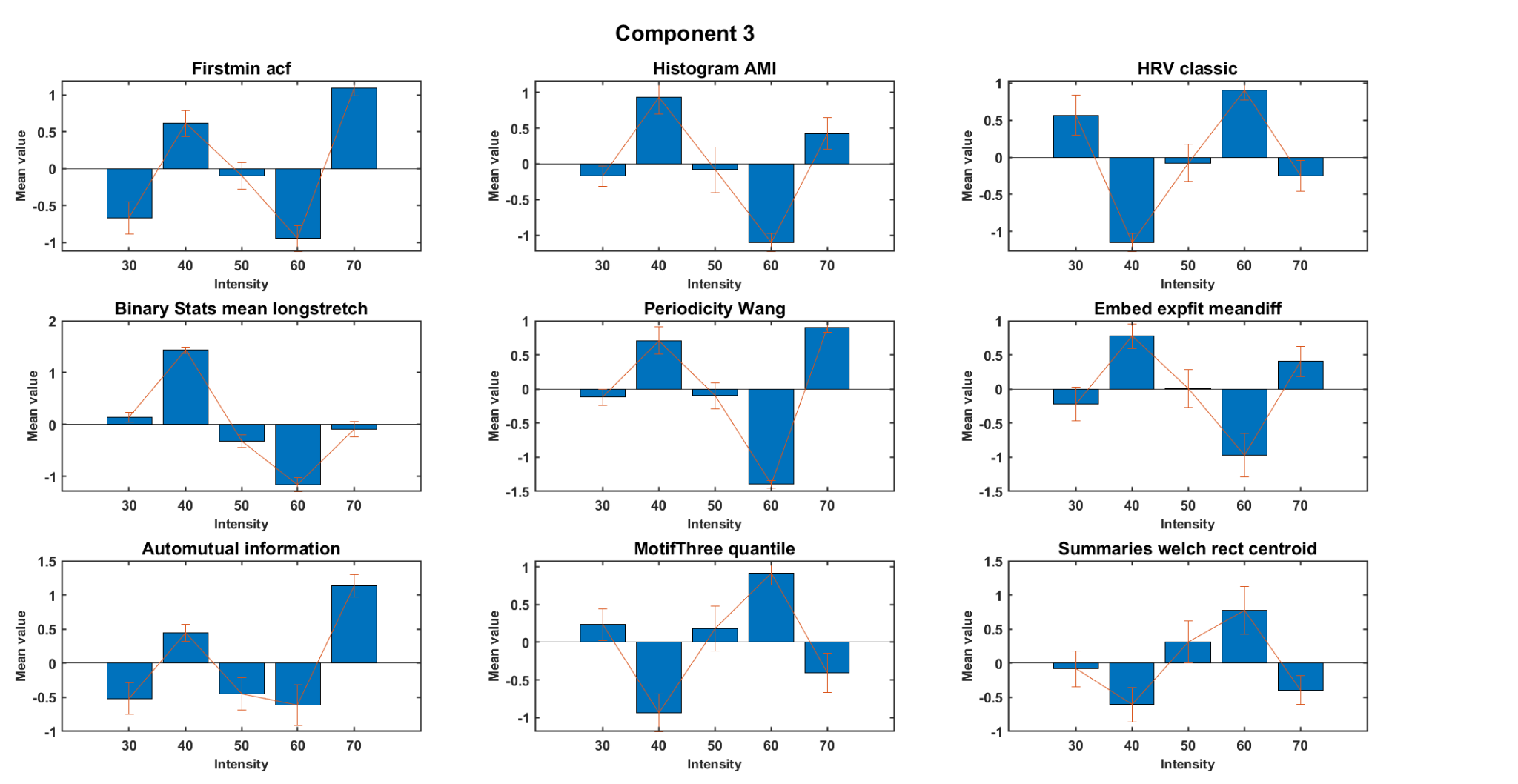


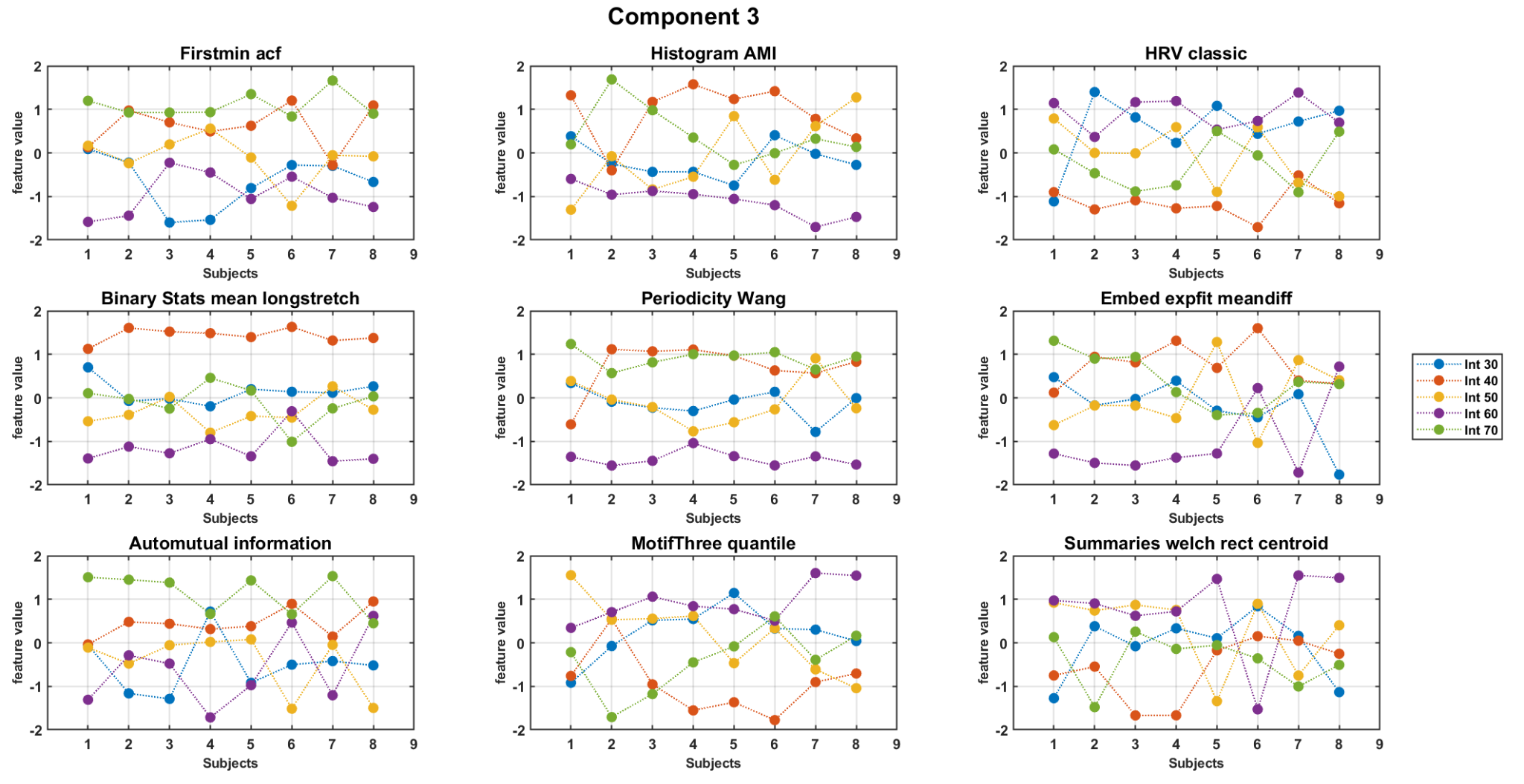


Fig. S1. Trend across intensity for features in component 3 for control group. The upper figure shows the aggregated (normalized) mean and standard deviation across subjects. The lower figure shows feature values for all subjects

Supplementary 3: We conducted two rounds of MCCA. In the first round, including 22 features, we analyzed Components 2–4 to identify most consistent features over subjects. Based on this analysis, we selected 9 features for the second MCCA. These features were then used to perform the second round of MCCA. Results of component 3 were brought in the main transcript, and components 2 and 4 are brought in fig S.2 and S.3.


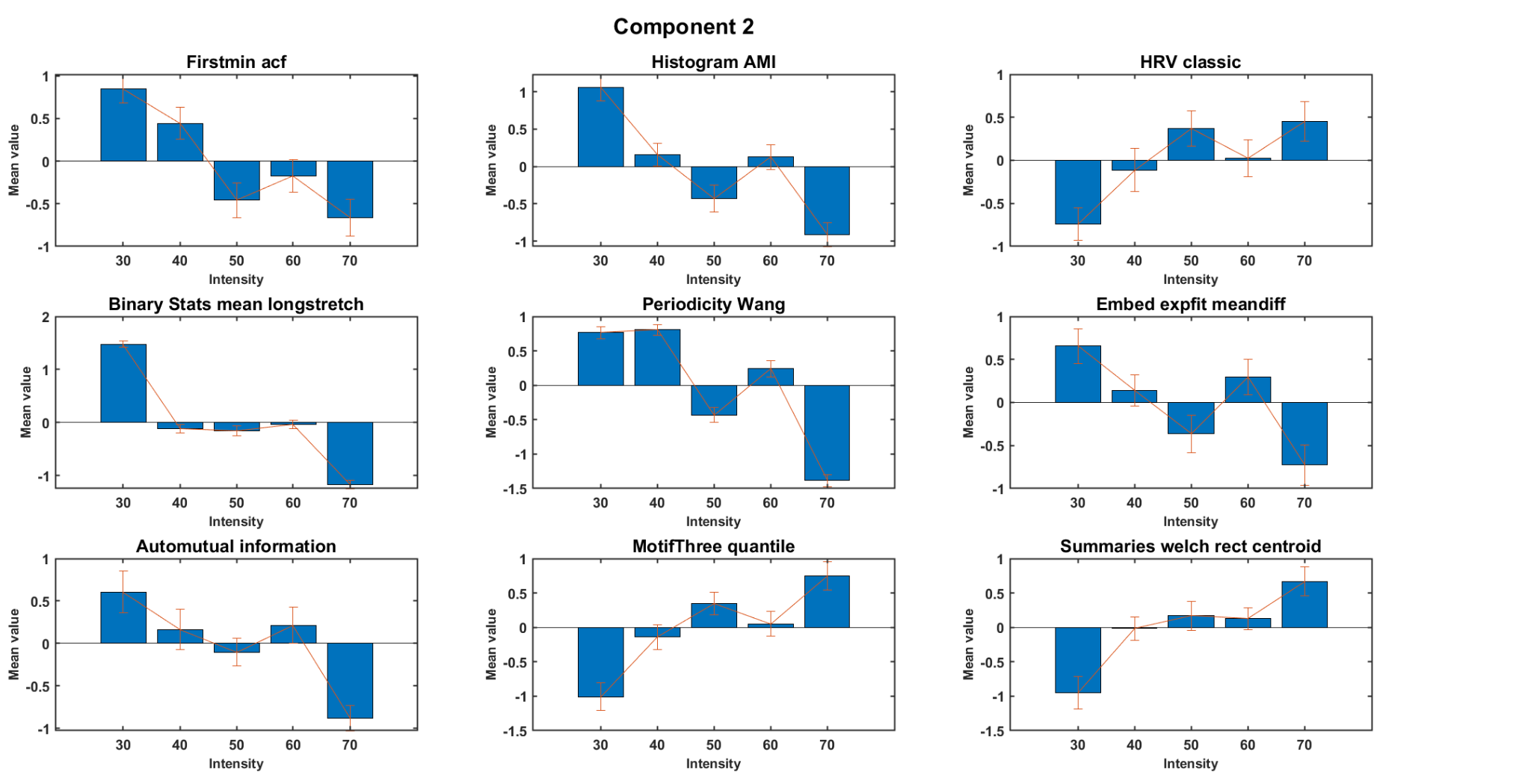


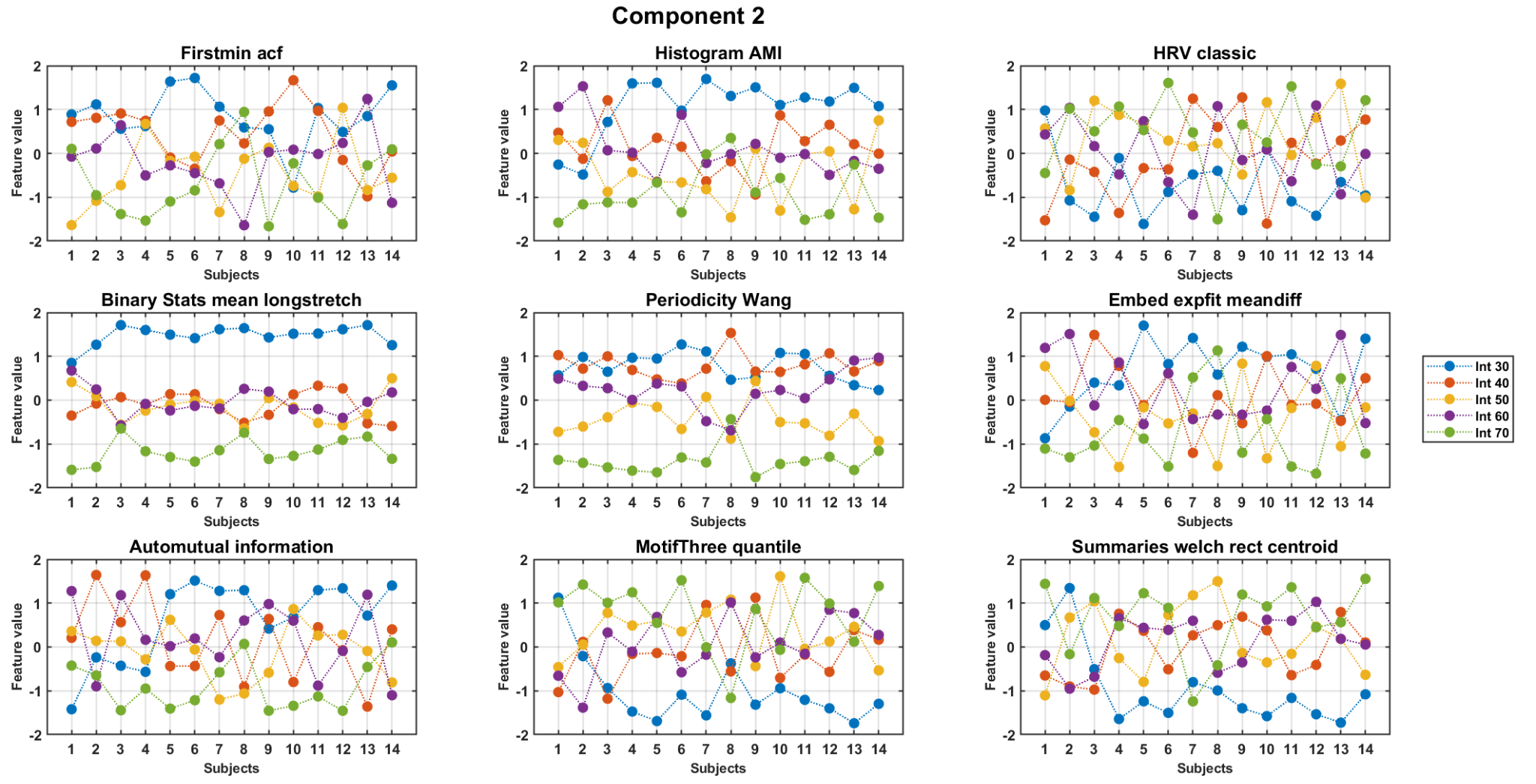


Fig. S2. Component 2 with the use of 9 selected features. Upper figure: displaying mean and variance over subjects in each intensity for each feature. Lower figure: displaying the result of the MCCA component for all subjects in each feature.


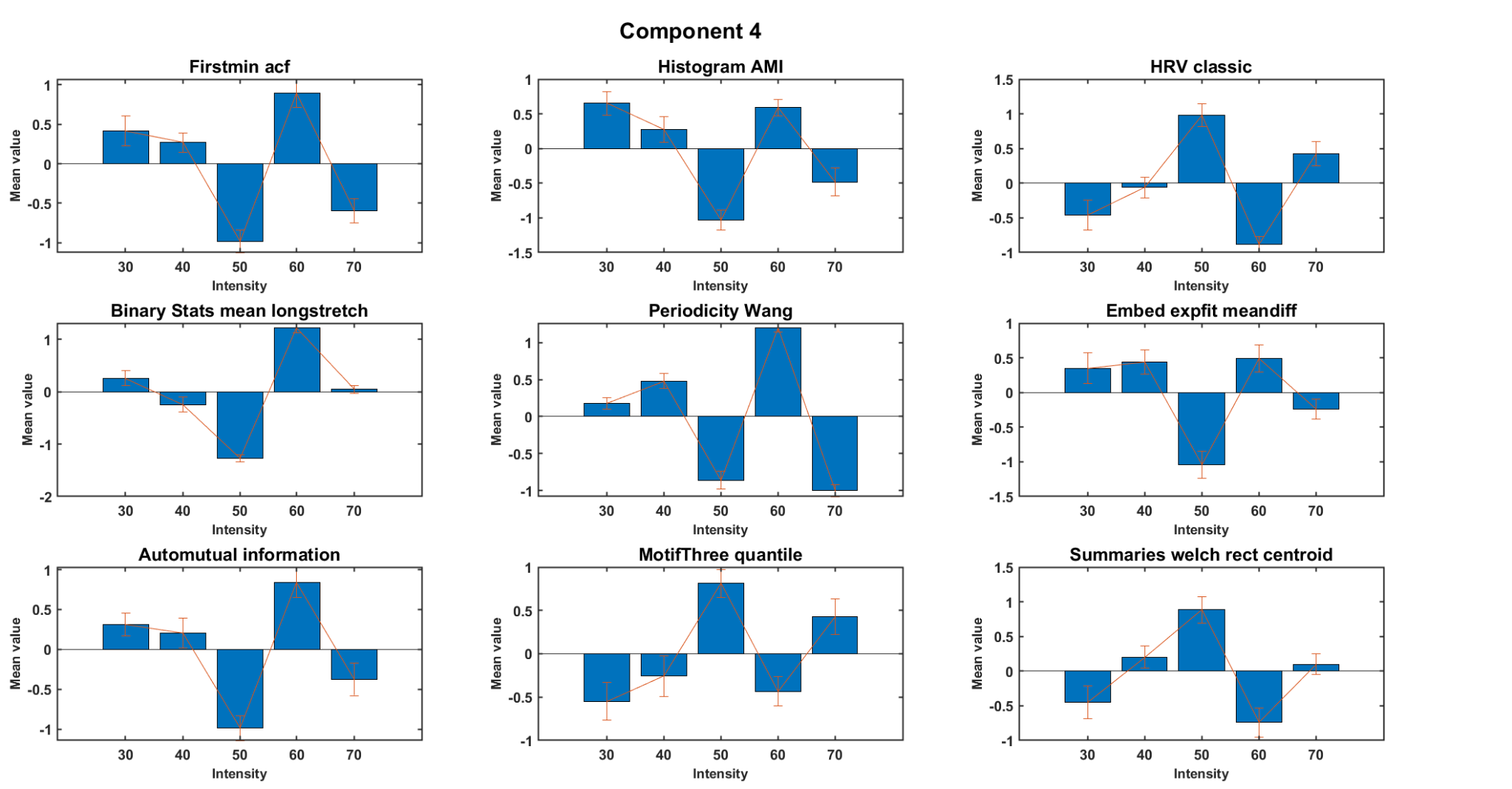


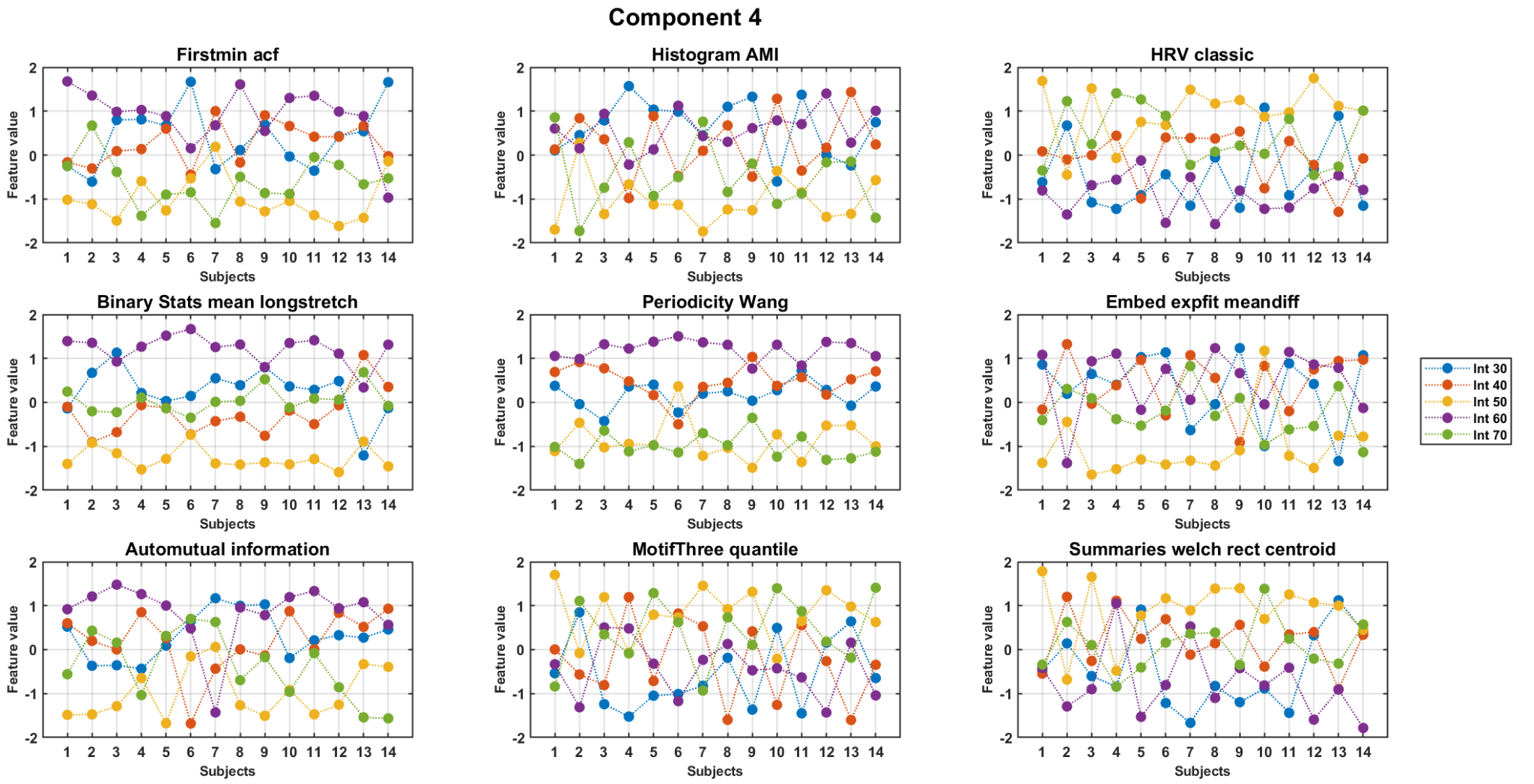


Fig S3. Component 4 with the use of 9 selected features. Upper figure: displaying mean and variance over subjects in each intensity for each feature. Lower figure: displaying the result of the MCCA component for all subjects in each feature.

## Supplementary 4: Using the score vector of component 3, we converted the matrix of EEG to a weighted vector. Results are brought in Fig. S4. The shift in dominant frequencies (of weighted averaged signal) from approximately 10 Hz at lower intensities to around 12 Hz at higher intensities demonstrates higher frequency components embedded at common patterns of higher pedaling intensity. This pattern was especially noticeable where the harmonic cluster changed frequency with an increase in intensity. The idea of higher periodicity at elevated intensities is supported by this shift, resulting in a longer time to detect the first peak in the autocorrelation function. This frequency shift suggests that increased physical effort demands more sustained neural processing, potentially reflecting the greater motor coordination required at higher exercise loads.


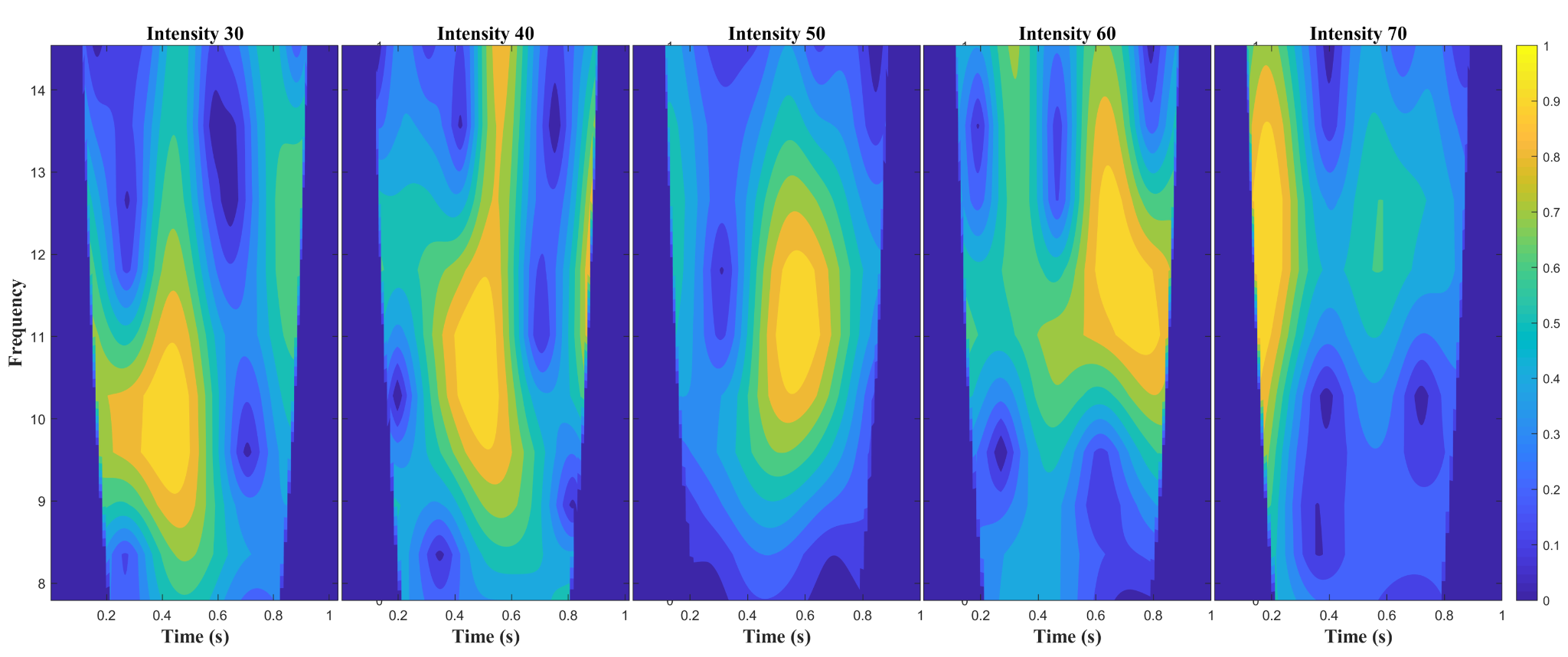


Fig. S4. Wavelet of the weighted EEG with MCCA scores for a selected subject, chosen for its clarity and ease of interpretation.

Supplementary 5: Results of post-hoc analysis for PD-HC comparison and intensity within PD comparison are brought in table S2.

Table S2: Post-hoc analysis for all 9 important features of component 3

| Feature | Comparison | F | pValue |
| --- | --- | --- | --- |
| FirstMin_acf | Intensity within PD | 135.19 | 0 |
|  | Intensity between PD and HC | 22.488 | 0.0001 |
| HistogramAMI_even_25 | Intensity within PD | 16.114 | 0.0006 |
|  | Intensity between PD and HC | 34.5 | 0 |
| Hrv_classic | Intensity within PD | 13.585 | 0.001 |
|  | Intensity between PD and HC | 21.145 | 0.0001 |
| BinaryStats_meanlongstretch | Intensity within PD | 11.787 | 0.002 |
|  | Intensity between PD and HC | 242.64 | 0 |
| Periodicity Wang | Intensity within PD | 514.5 | 0 |
|  | Intensity between PD and HC | 533.77 | 0 |
| Embed2_Dist_expfit_meandiff | Intensity within PD | 21.445 | 0.0001 |
|  | Intensity between PD and HC | 35.038 | 0 |
| AutoMutualInfoStat | Intensity within PD | 77.027 | 0 |
|  | Intensity between PD and HC | 8.7919 | 0.007 |
| MotifThree_quantile | Intensity within PD | 10.25 | 0.004 |
|  | Intensity between PD and HC | 18.839 | 0.0003 |
| LocalSimple_mean3stderr | Intensity within PD | 11.815 | 0.002 |
|  | Intensity between PD and HC | 24.298 | 0 |
